# Supplementary figures and images for: Two opposing gene expression patterns within ATRX aberrant neuroblastoma
Source: PLoS One. 2023 Aug 4;18(8):e0289084. doi: 10.1371/journal.pone.0289084 (PMC10403137; doi:10.1371/journal.pone.0289084)

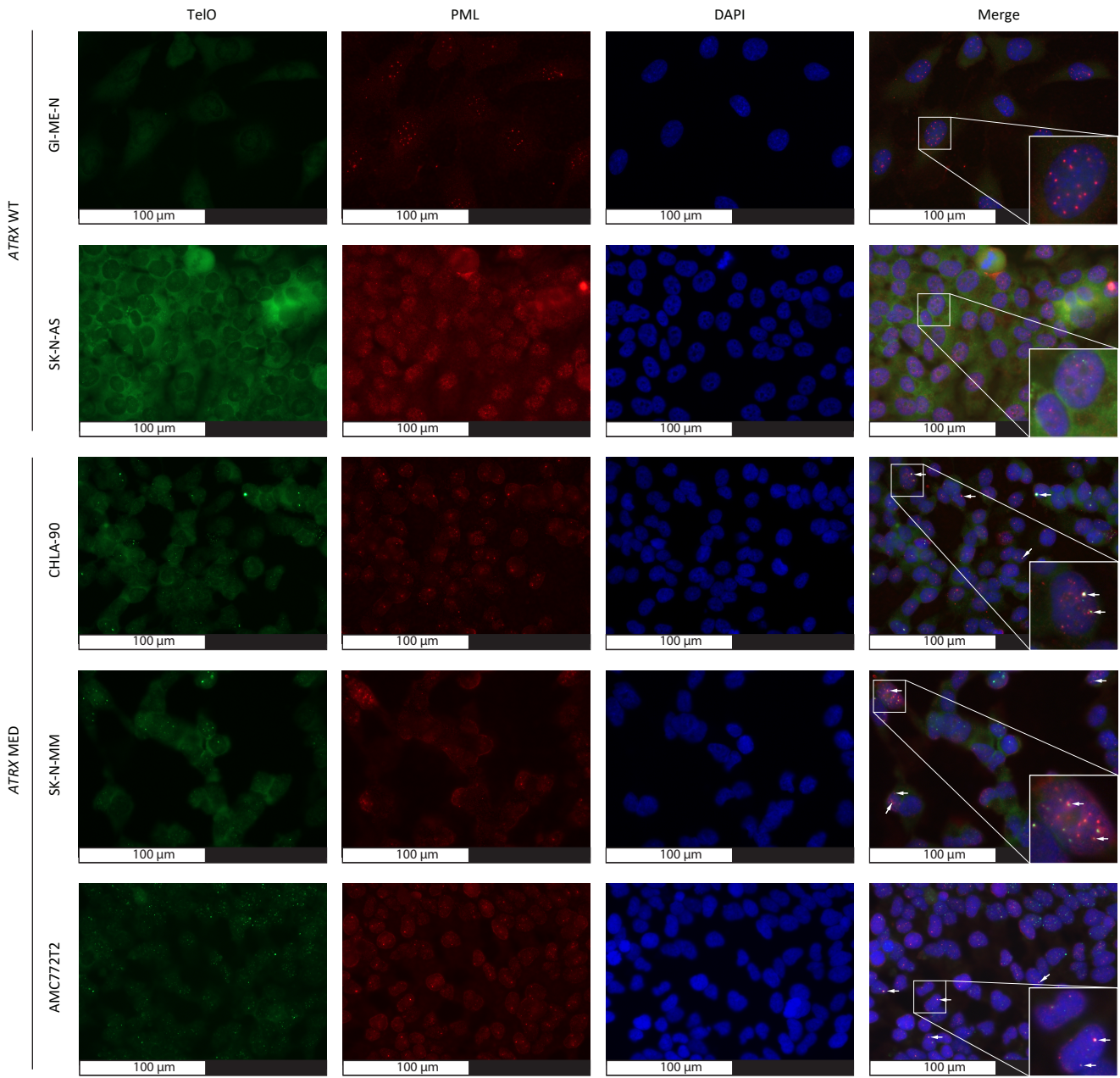

Supplement: S1 Fig — White arrows mark co-localisation of telomeric (TelO) and PML foci, only a maximum of four arrows per image is displayed. (PDF) [file pone.0289084.s001.pdf]

**A**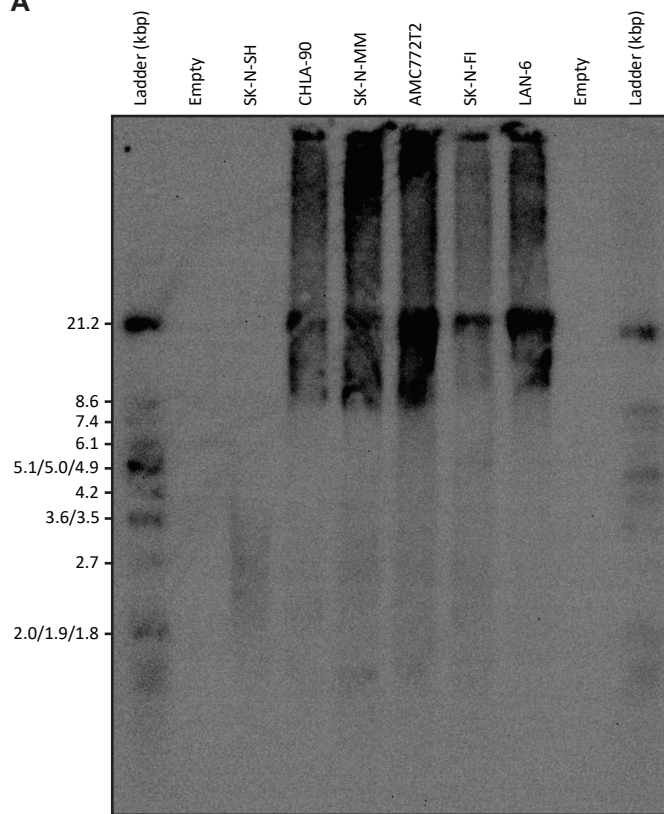**B**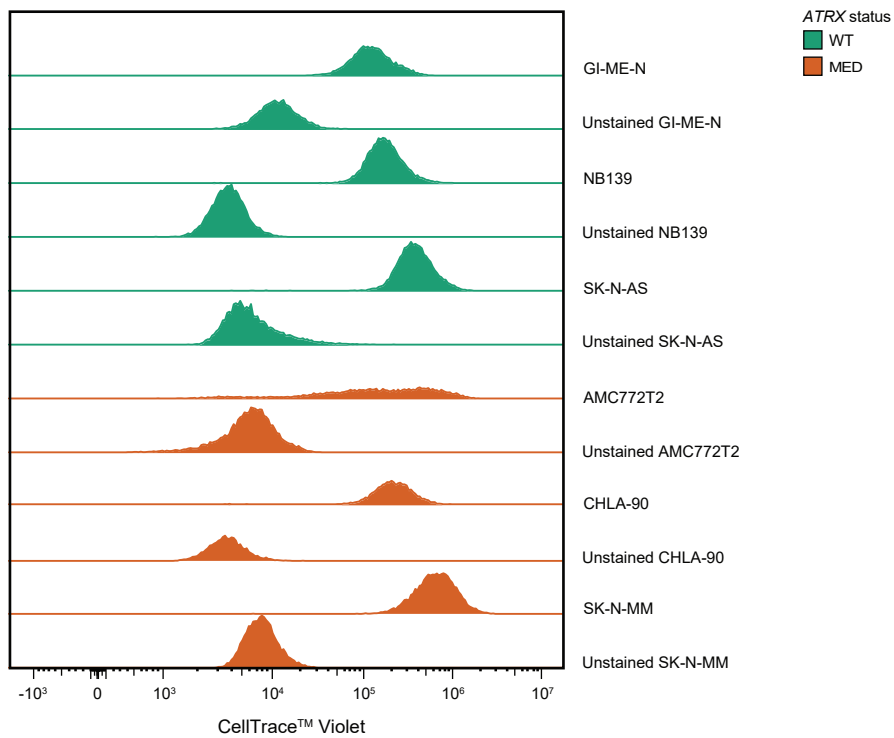

Supplement: S2 Fig — (a) Southern blot containing one ALT negative cell line (SK-N-SH) and two well-known ALT positive neuroblastoma cell lines (SK-N-FI and LAN-6). All three PDΔATRX models display long and heterogeneous telomeres and therefore confirm ALT. (b) Violet trace experiments on three ATRXWT and on three PDΔATRX models. (PDF) [file pone.0289084.s002.pdf]

**A**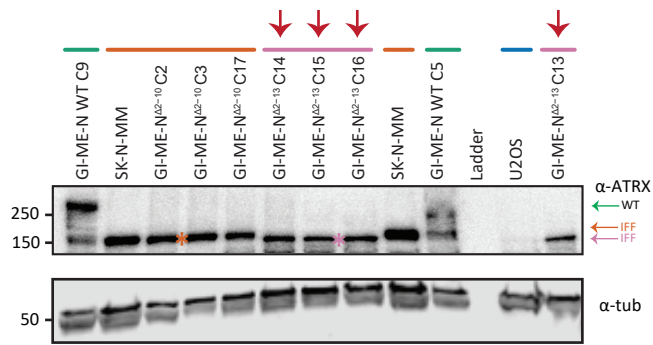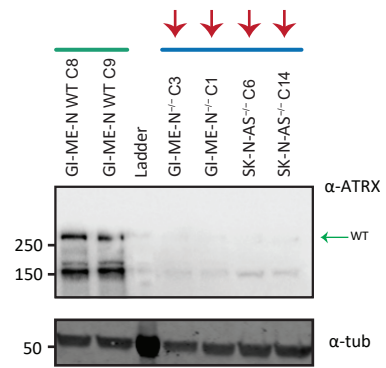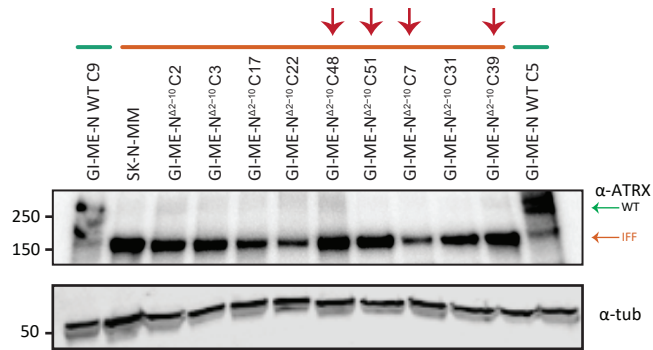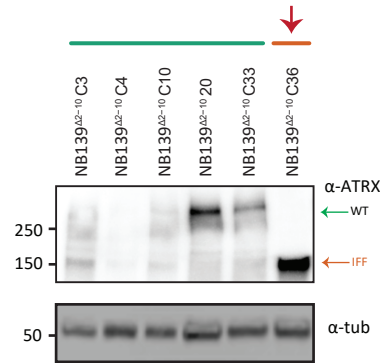**B**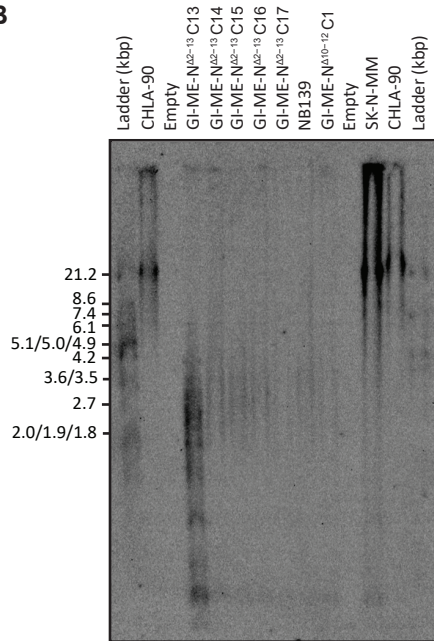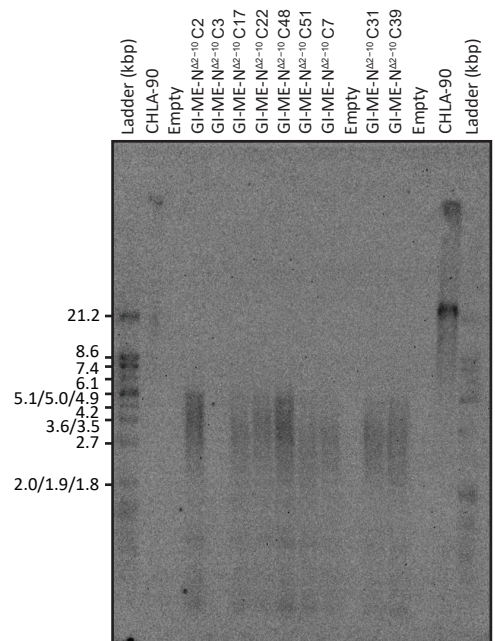

Supplement: S3 Fig — (a) Western blots for ATRX protein of all the correct ATRX aberrant clones. Clones that were send for sequencing are marked with a red arrow on top. Green bar: confirmed wild-type clones, dark orange bar: confirmed ATRXΔ2–10 clones or PDΔATRX models, pink bar: confirmed ATRXΔ2–13 clones and dark blue bar: confirmed ATRX-/- clones or the ATRX-/- osteosarcoma cell line U2OS. IFF: ATRX in-frame fusion protein product. Dark orange and pink asterisks indicate ATRX exon 2–10 and exon 2–13 MED IFF protein products, respectively. Stainings against α-tubulin were used as reference. (b) Southern blots confirming absence of long heterogeneous telomeres in our isogenic ATRX aberrant models. CHLA-90 and SK-N-MM were used as positive controls. (PDF) [file pone.0289084.s003.pdf]

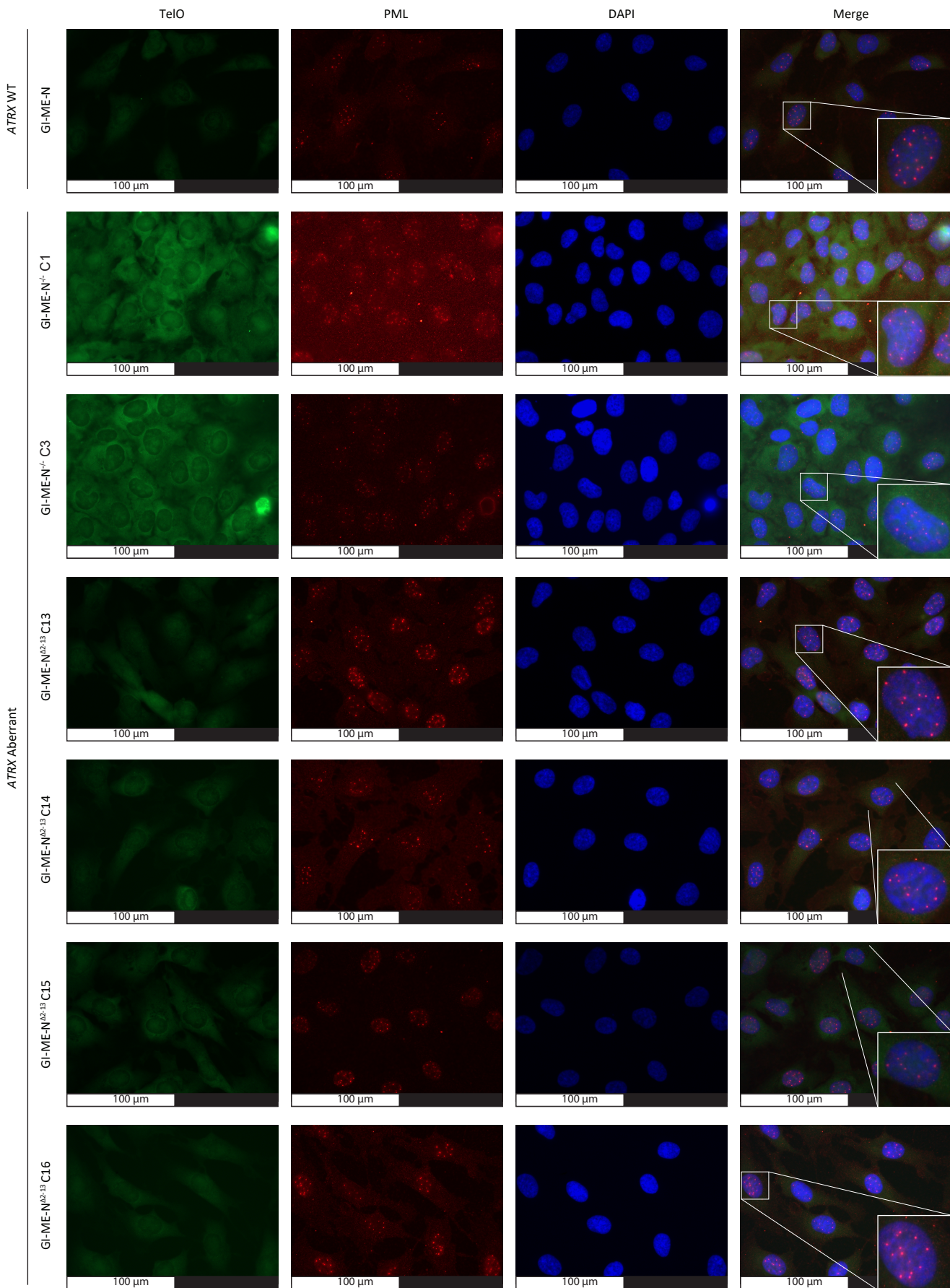

Supplement: S5 Fig — (PDF) [file pone.0289084.s005.pdf]

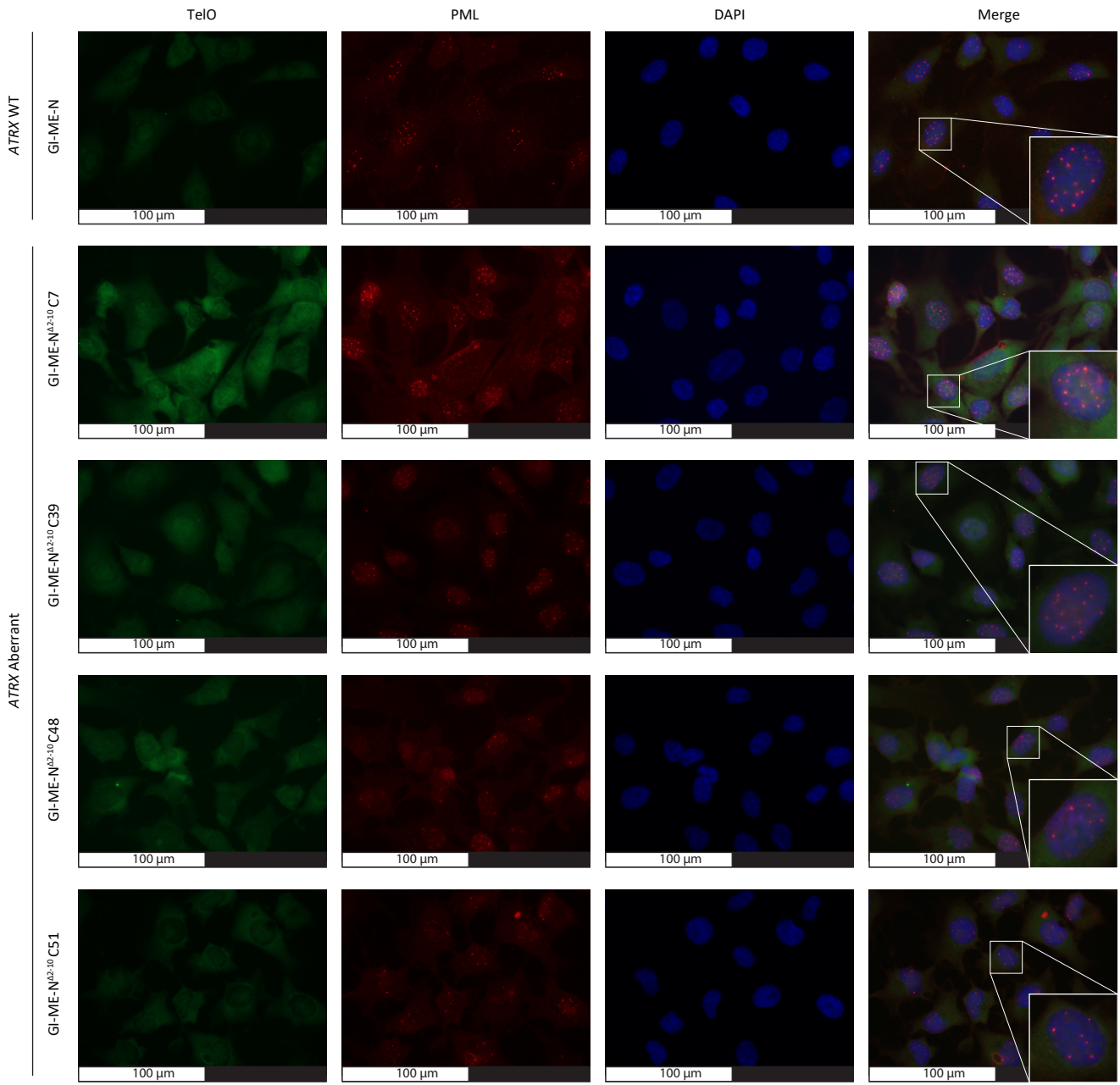

Supplement: S6 Fig — (PDF) [file pone.0289084.s006.pdf]

**A**

GI-ME-N

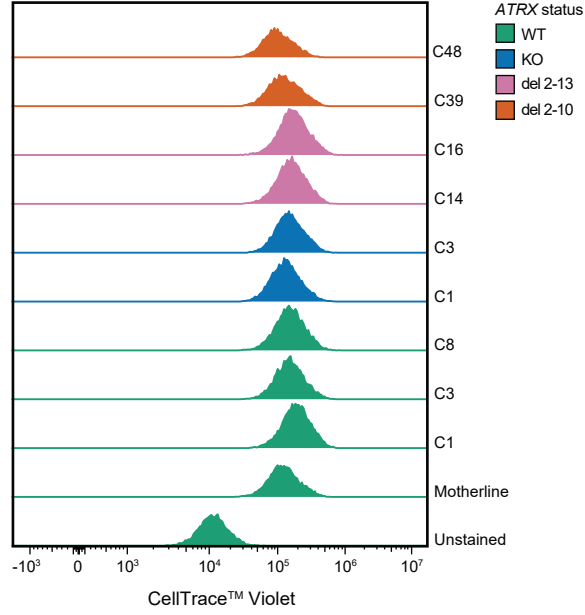**B**

NB139

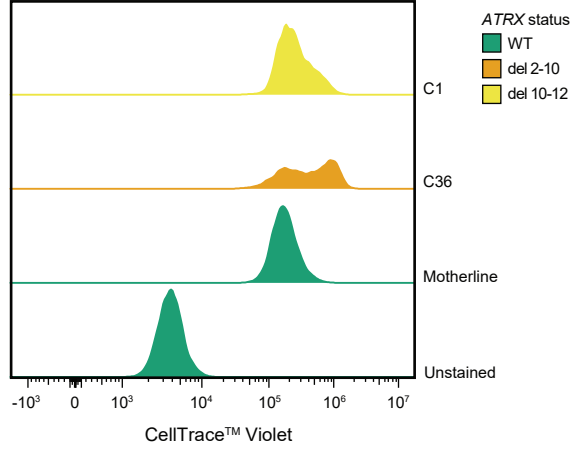**C**

SK-N-AS

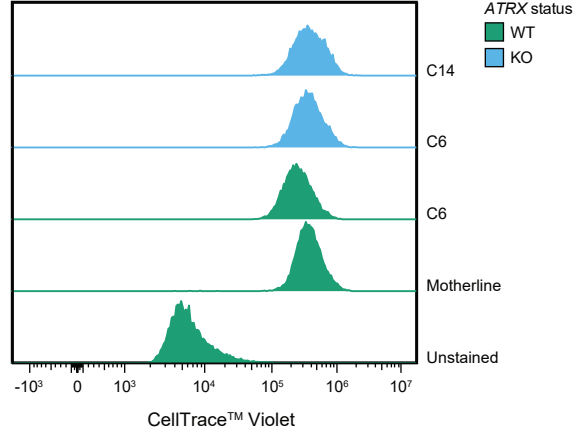

Supplement: S7 Fig — (a-c) Violet trace experiments on ATRX wild-type and isogenic ATRX aberrant (a) GI-ME-N, (b) NB139 and (c) SK-N-AS models. (PDF) [file pone.0289084.s007.pdf]

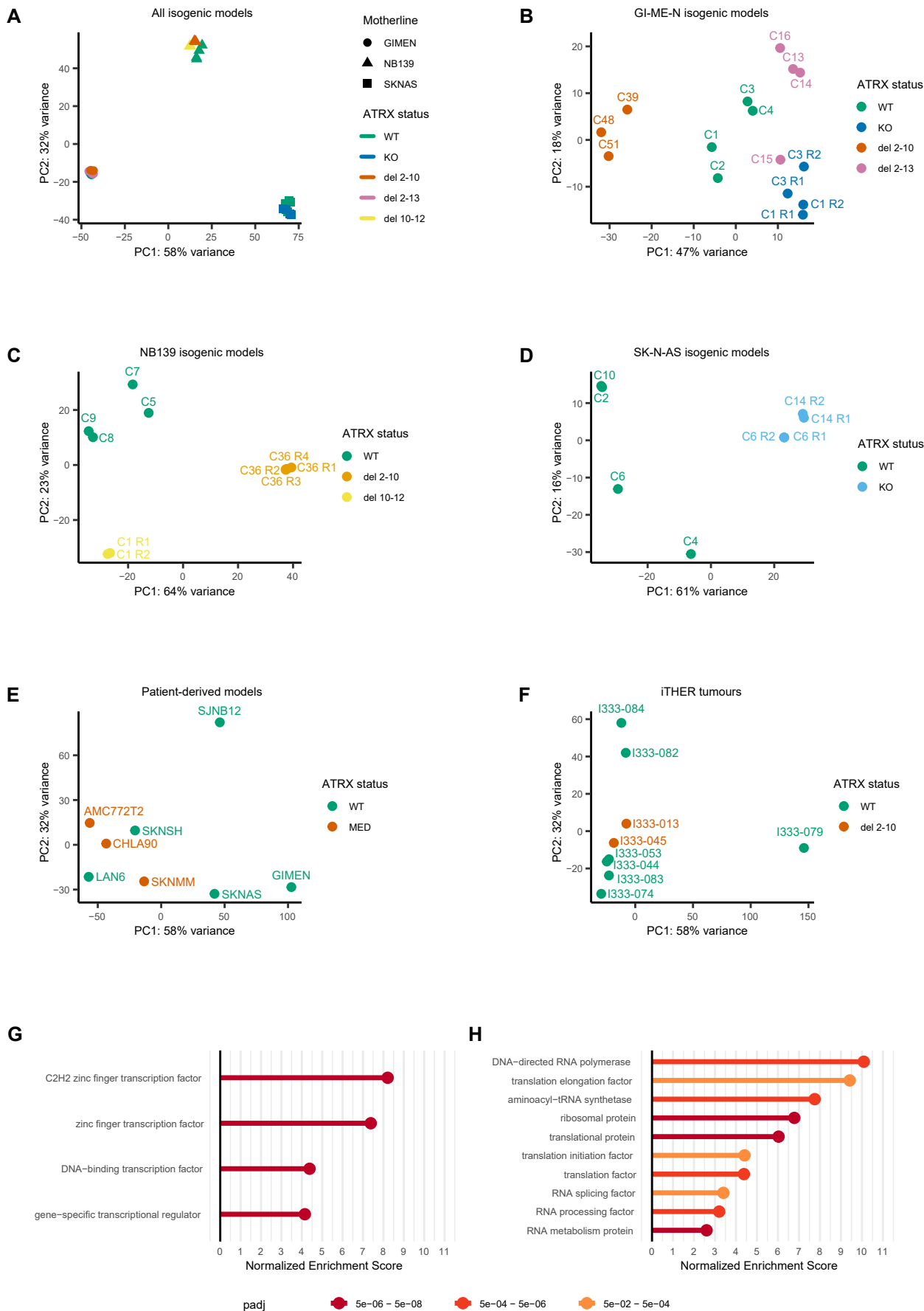

Supplement: S8 Fig — (a) PCA of all generated isogenic ATRX aberrant clones showing separation based on the mother-lines. (b-f) PCA of (b) isogenic GI-ME-N clones, (c) isogenic NB139 clones, (d) isogenic SK-N-AS clones, (e) patient-derived ATRX aberrant and wild-type models (f) ATRX aberrant and wild-type iTHER tumours. (g-h) Significantly enriched Panther protein classes of the overlapping differentially expressed downregulated genes (g) for all ATRX-/- and ATRXΔ2–13 isogenic models and (h) for all ATRXΔ2–10 isogenic models. (PDF) [file pone.0289084.s008.pdf]

# GO analysis overlapping downregulated genes deletion exon 2-10 models

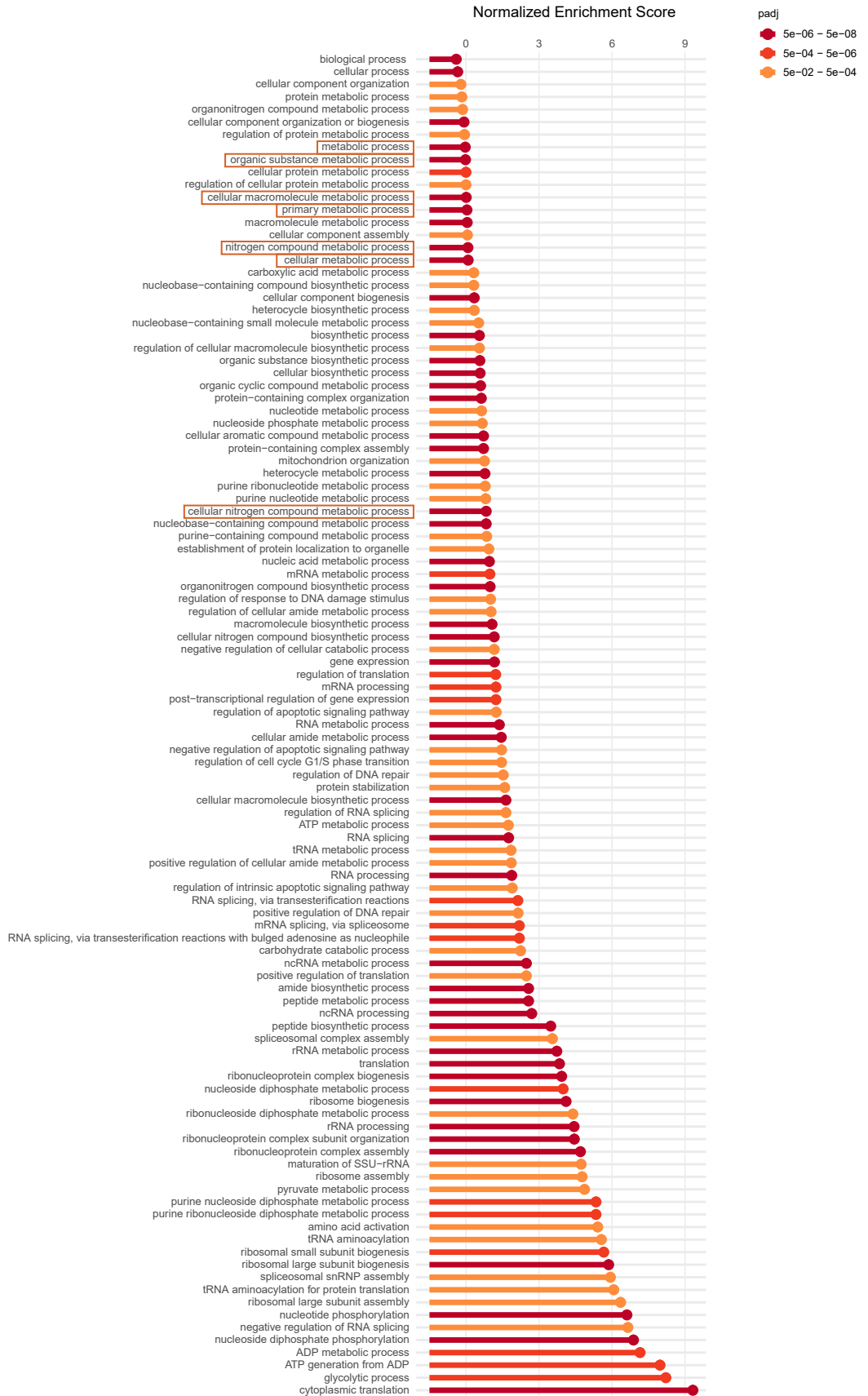

Supplement: S9 Fig — Orange boxes highlighted the same terms as observed in Fig 3E. (PDF) [file pone.0289084.s009.pdf]

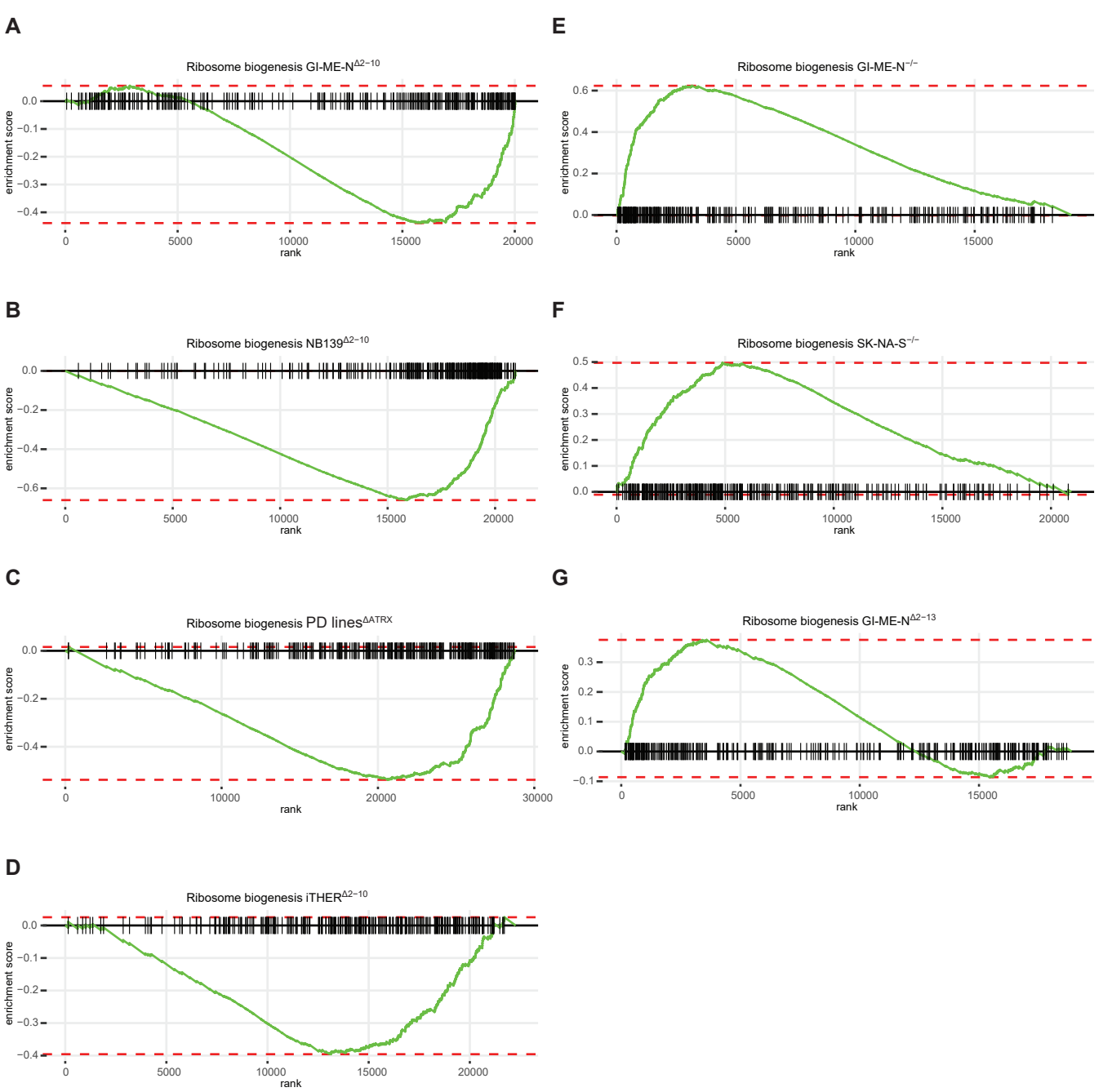

Supplement: S10 Fig — Ribosome biogenesis enrichment plots for (a) GI-ME-NΔ2–10 (b) NB139Δ2–10 (c) PDΔATRX (d) iTHERΔ2–10 (e) GI-ME-N-/- (f) SK-N-AS-/- (g) GI-ME-NΔ2–13. (PDF) [file pone.0289084.s010.pdf]

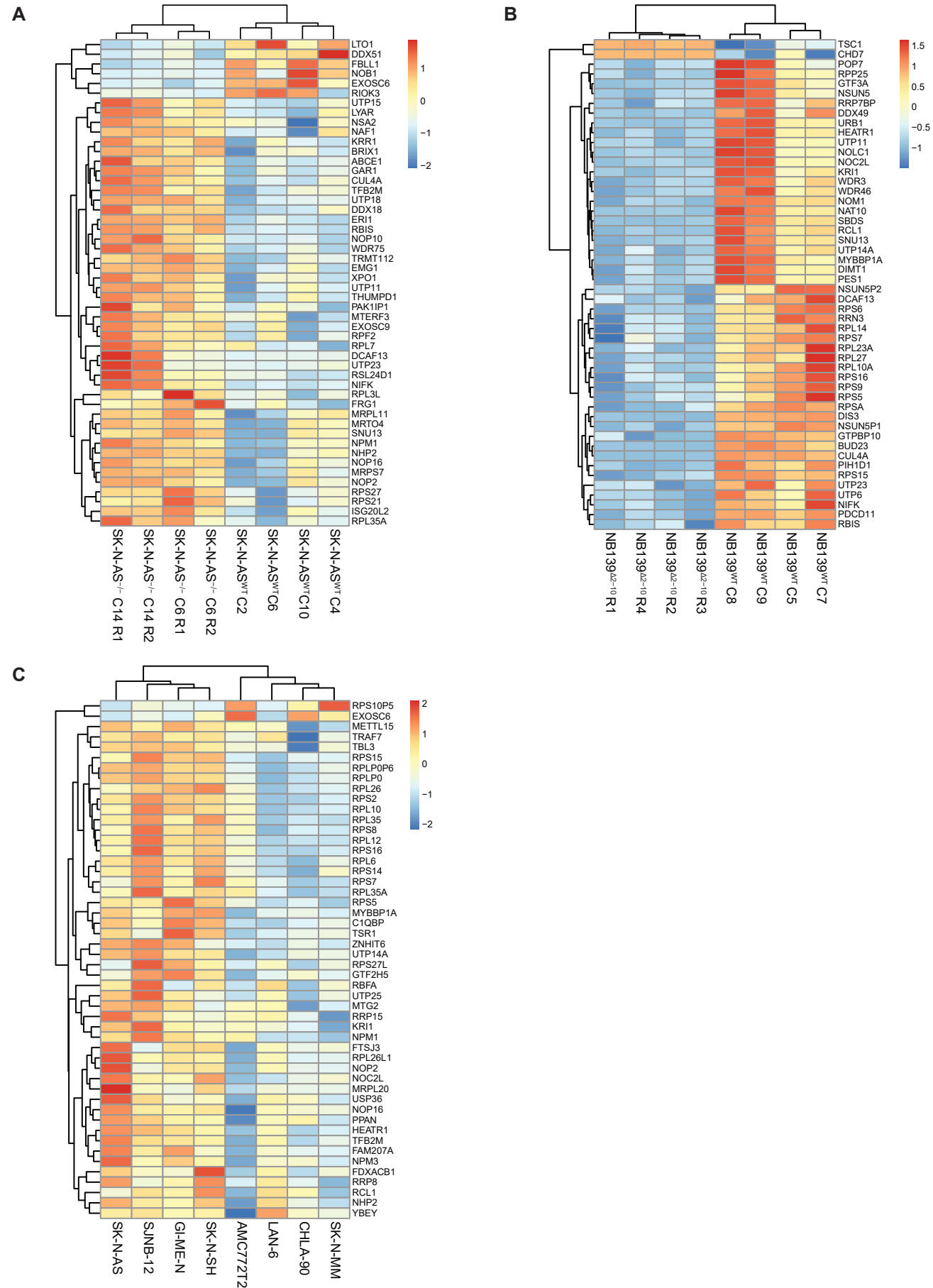

Supplement: S11 Fig — (a-c) Heatmaps showing expression values for the top 50 differentially expressed ribosome biogenesis genes that are normalized across all samples by Z-score in (a) SK-N-AS-/- (b) NB139Δ2–10 and (c) PDΔATRX. Both row and column clustering were applied using the Euclidean distance. (PDF) [file pone.0289084.s011.pdf]

**A**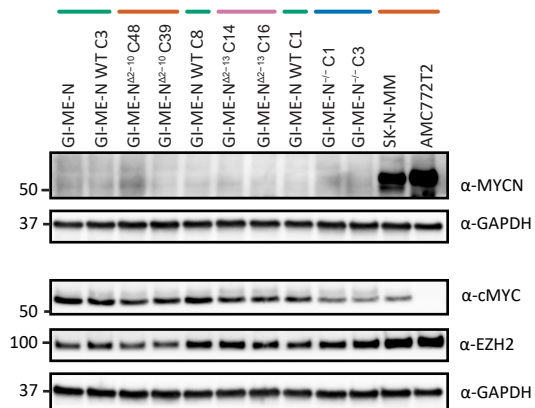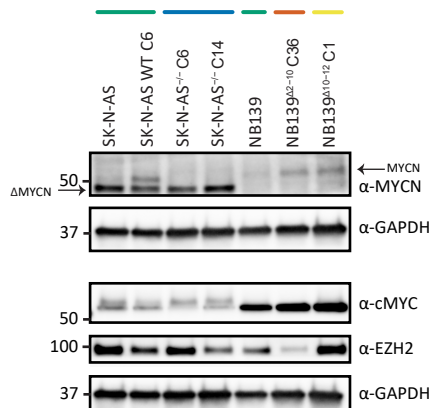**B**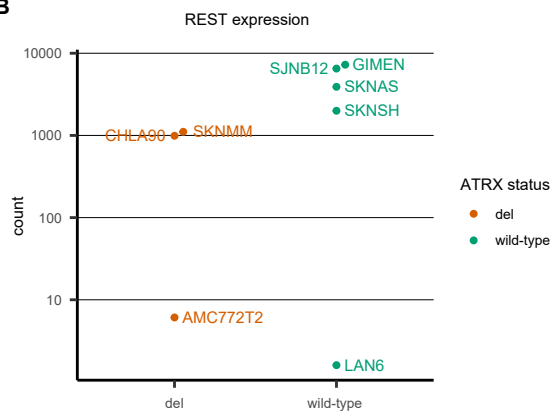**C**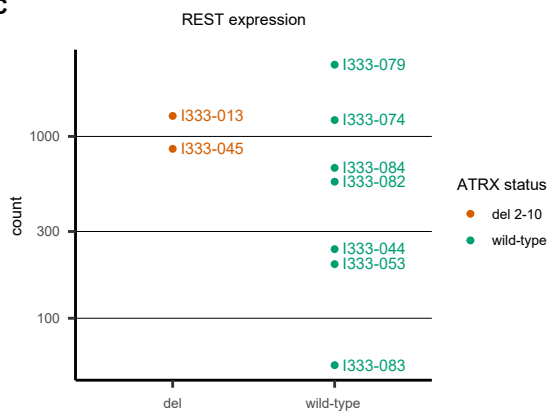**D**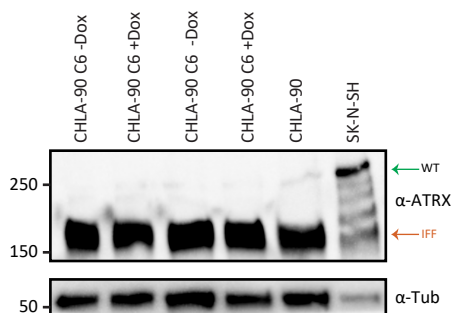**E**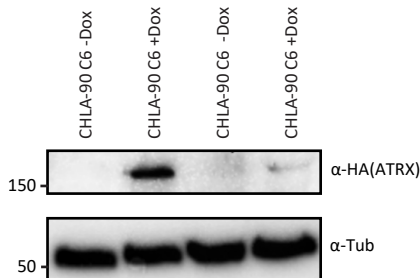

Supplement: S12 Fig — (a) Western blot analysis for three proteins (MYCN, cMYC and EZH2) that are directly involved in regulating ribosome biogenesis revealed no changes that are consistent with our identified expression pattern dichotomy within ATRX aberrant models. Two distinct isoforms of MYCN are displayed in the right blot, with SK-N-AS being the only model expressing ΔMYCN. The color bars on top correspond to the ATRX status (green: wild-type, dark orange: exon 2–10 MED (or exon 2–9 in PDΔATRX models), blue: knock-out, pink: exon 2–13 MED and yellow: exon 10–12 MED). Stainings against GAPDH were used as reference. (b) REST gene expression in patient-derived ATRX MED and wild-type models. (c) REST gene expression in ATRX aberrant and wild-type iTHER tumours. (d) Western blot analysis showing unchanged ATRX IFF protein expression upon doxycycline induction of ATRXWT protein expression. (e) Western blot analysis showing presence of HA-tagged ATRXWT protein product only upon induction with doxycycline. (d-e) Stainings against α-tubulin were used as reference. (PDF) [file pone.0289084.s012.pdf]
