# Supplementary material for: Two opposing gene expression patterns within ATRX aberrant neuroblastoma
Source: PLoS One. 2023 Aug 4;18(8):e0289084. doi: 10.1371/journal.pone.0289084 (PMC10403137; doi:10.1371/journal.pone.0289084)
Supplement: S4 Fig — White arrows mark co-localisation of telomeric (TelO) and PML foci, only a maximum of four arrows per image is displayed. CHLA-90 was added as positive control. (PDF) [file pone.0289084.s004.pdf]

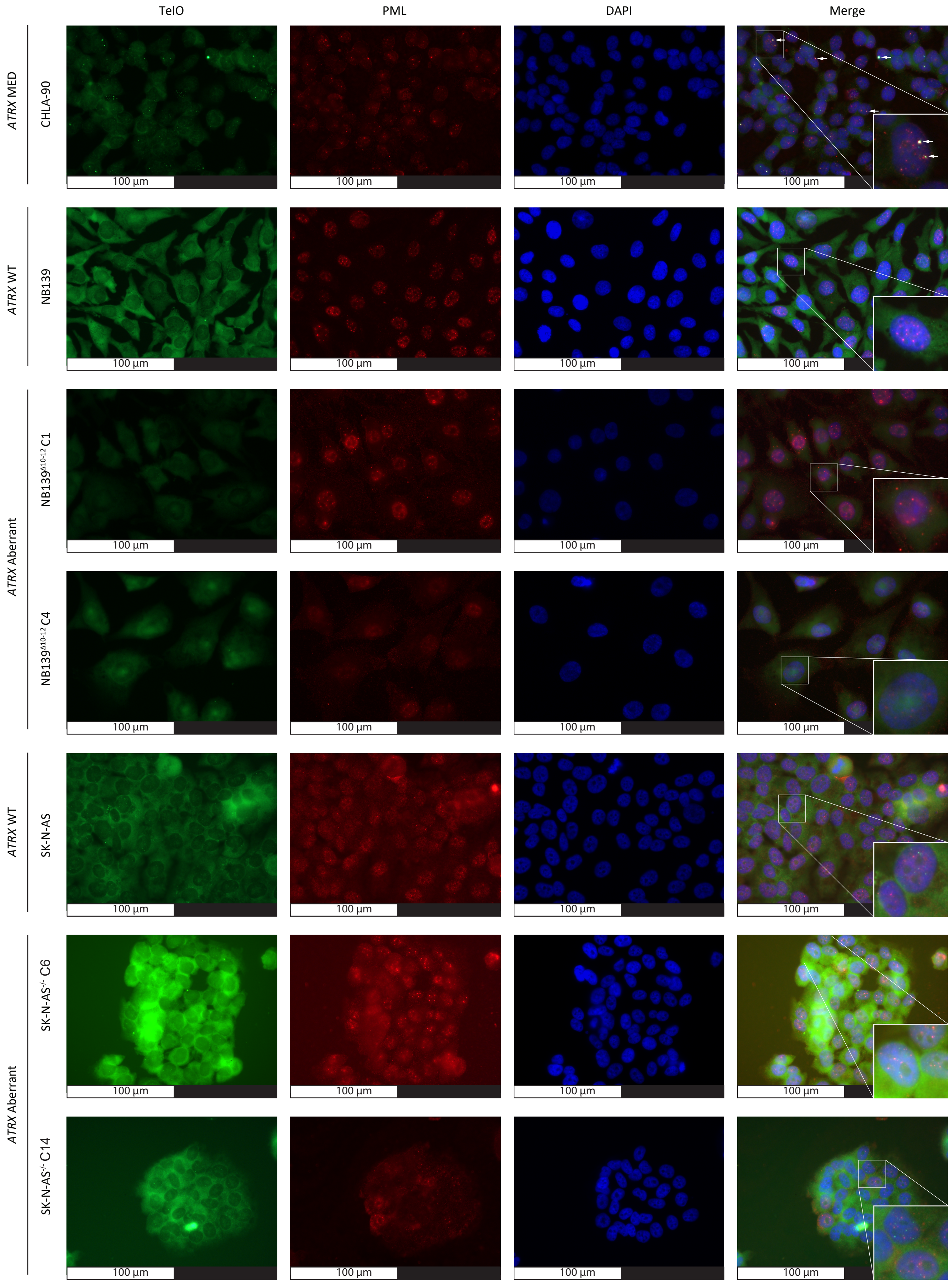
TelO
PML
DAPI
Merge
ATRX MED
CHLA-90
ATRX WT
NB139
ATRX Aberrant
NB139Δ10-12 C1
NB139Δ10-12 C4
ATRX WT
SK-N-AS
ATRX Aberrant
SK-N-AS-/- C6
SK-N-AS-/- C14
100 µm
