# Supplementary material for: Two opposing gene expression patterns within ATRX aberrant neuroblastoma
Source: PLoS One. 2023 Aug 4;18(8):e0289084. doi: 10.1371/journal.pone.0289084 (PMC10403137; doi:10.1371/journal.pone.0289084)
Supplement: Attachment — Submitted filename: revision-plan_and_response_to_reviewers_updated.1.docx [file pone.0289084.s021.docx]

**Manuscript number:** RC- 2022-01741

**Corresponding author(s):** Marlinde L. van den Boogaard

## 1. General Statements [optional]

*We would like to thank the reviewers for their constructive comments, and we tried to revise the manuscript accordingly.*

## 2. Description of the planned revisions 🡪 these are now also incorporated in the manuscript.

***Reviewer 1***

Part of major comment 1. Unfortunately, not all the claims made are adequately supported by the data presented. In many experiments, the number of biological replicates is insufficient (sometimes n=1). This would have to be remedied prior to publication, to ensure the data can be properly interpreted.

The reviewer is specifically referring to our cell cycle analysis (as these are the only experiments where some of them only contain one replicate; e.g., figure 1D and figure 2E).

In the previous months we performed for each biological replicate two additional technical replicates to increase the number of measurements. Additionally, we also performed three technical replicates for an additional GIMEN ATRX exon 2-10 clone and three technical replicates for two additional GIMEN ATRX exon 2-13 clones. Also, for SKNAS (Figure 2F) we performed measurements for two more wildtype clones in triplicate. This also increased the number of biological replicates where possible.

***Reviewer 1***

Major comment 5. More generally, the data is presented in a disorganized way, making it difficult to follow. Some are in main figures, some in supplementary, some experiments are done on only a subset of clones (i.e. cytoplasmic vs nuclear distribution). The authors should try to show all relevant results (for example western, facs data) for all their lines in the main figure, so that they can be compared, with adequate number of replicates and statistical analysis.

To improve on these points raised by the reviewer, we performed additional cell cycle analyses to get an adequate number of replicates and we performed statistical analyses. We also added the cell cycle analysis of SKNAS (old figure S5A) to main figure 2 and we added the western blot for yH2A.X of SKNAS and NB139 (old figure S4C) to main figure 2. Regarding the cytoplasmic vs. nuclear distribution, we now also included a fractionation experiment for the

NB139 models. We won’t perform these experiments for the SKNAS models since they are ATRX knockout.

***Reviewer 3***

Major comment 4. In Figure 2D-E, cell cycle analysis was performed with ATRX WT and multi-exon ATRX deletions and there is an increased percentage of cells visible in the S phase compared to WT cells. Still, it is not clear from the Figure whether the result is statistically significant. The experiment should be repeated one more time and a statistical test should be done.

These comments were also postulated by reviewer 1. We increased the number of measurements for our FACS experiments and included the statistical analysis (for more detail see the response to the comments of reviewer 1).

***Reviewer 3***

Part of major comment 6. Statistical analyses is missing from almost every Figure.

As mentioned above we included the missing statistical analyses on the cell cycle analysis. Due to this comment, we noticed that we forgot to include the text about the statistical analyses in the legend of figure 5 (the p-values and statistics were mentioned in the result section), which we now changed in this revision.

## 3. Description of the revisions that have already been incorporated in the transferred manuscript

***Reviewer 1***

Part of major comment 1. Each data point should be indicated in bar graphs (for example in Figure 5, especially given the variability observed).

In figure 5 we now added the data points in the figures.

***Reviewer 1***

Major comment 2. The Western blot data is often very difficult to interpret, given that many bands are present in addition to the specific ones for the WT and FTT bands. Even for some controls presented as WT, the full-length protein is very faint while other bands predominate. This should be explained in the text. If no explanation is available, I would recommend confirming the results with other ATRX antibodies.

There are several other known isoforms of ATRX, namely around 250, around 200 and 150 kDa, we now mention them in the text (page 4) and in the main and supplementary figures we made the panels smaller (figure 1B and S3A), to remove all the non-important bands below the IFFs. A possible explanation for the faint full-length bands is that the mutant *ATRX* protein products are much stronger expressed and therefore during blot development the wild-type bands become faint. Previously, we already tested other ATRX antibodies, and they either showed similar patterns in bands (also many bands observed) or performed much worse.

***Reviewer 1***

Major comment 3. While the Western blot data suggests that ATRX protein products from MEDs are largely retained in the cytoplasm, this is not observed in the immunofluorescence pictures shown in supplementary figures. The authors should make a decision whether to provide more convincing and clear data, or to remove the immunofluorescence data.

We agree with the reviewer that the ATRX fractionation western blot have superior resolution over the stainings and therefore we decided to remove these stainings from the manuscript.

***Reviewer 1***

Major comment 4. The immunofluorescence data shown in supplementary figures are not of adequate quality. It is impossible to see much of what the authors are claiming. The Telomere and PML images are especially problematic.

We agree with the reviewer that without zooming in in the word file it might be hard to detect the co-localizations for the telomere and PML images. To resolve this, we made zoom-ins for single cells for the merged panels (only for CHLA-90, SK-N-MM and AMC772T2 co-localization can be observed; many studies only use the TelO staining as telomeric dots are often exclusively observed in ALT lines, however sometimes false positive can be observed and therefore including PML reduces the rate of false positives). Additionally, we also performed southern blots, which confirmed the telomere and PML stainings.

***Reviewer 1***

Minor comment 1. Some grammatical errors should be corrected throughout.

We re-read the entire manuscript and changed the grammatical mistakes that we could detect.

***Reviewer 1***

Minor comment 2. Supplementary Table 1 was mislabeled as "Supplementary Figure 1"

We corrected this.

***Reviewer 1***

Extra comment. *The authors should comment on the differences between the protein products (MED exon 2-10 vs MED exon 2-13) that could cause opposite transcriptional effects. What are the protein motifs that will be affected in one but not the other, and could this explain different effects on transcription, especially considering their claim that the majority of these protein products remain in the cytoplasm.*

*We added a paragraph to the discussion addressing this (page 18), but unfortunately no domains are currently known for the region of exon 11-13. Our claim that the majority of the protein resides within the cytoplasma is supported by the paper Qadeer et al., 2019.*

***Reviewer 2***

Minor comment 1. Can the authors generalize these observations to other cancers with ATRX mutations?

In our discussion, we already mentioned increased ribosome biogenesis in glioma tumors with nonsense mutations, but we have included an additional sentence about these observations after that sentence (page 18).

***Reviewer 3***

Major comments 1. In Figures 3 and 4, the authors showed two distinct gene set enrichment profiles in the ATRX deletion constructs ATRXΔ2-13 and ATRXΔ2-10. They used GI-ME-N WT clones C1 and C2 for Figure 4D, whereas in Figure 4E, they utilized WT clones C3 and C4. It is not clear from the above two Figures how WT C1, C2 are different with WT GI-ME-N C3 and C4 and share distinct gene signatures. The authors should put the supplementary Figure 15A into the main Figure 4 and use the same WT GI-ME-N clones while comparing the gene expression with ATRX KO or ATRXΔ2-13, or ATRXΔ2-10. Is the difference in gene signature between ATRXΔ2-13 and ATRXΔ2-10 due to the heterogeneity present in the WT GI-ME-N cells?

This might indeed be confusing. In our material and methods section, we addressed this under header: RNA sequencing analysis. Here we mentioned: “For the GI-ME-N clones, we observe a batch effect in the wild-type clones. Therefore, we decided to compare the different GI-ME-N ATRX aberrant models only with their corresponding wild-type clones (generated by same person).” To make this more clear, we now mention this in the result section (page 9). Our GI-ME-N models were generated in two batches (each batch by a different person, while the harvest and work-up of the RNA samples was performed by the same person on the same day for all samples) and therefore we decided to send two wild-type clones belonging to one batch and two clones belonging to the other batch (wildtype clone 3 and 4 were created by the same person as the GI-ME-N ATRXΔ2-10, while clone 1 and 2 were created by the same person as all the other GI-ME-N models). In our PCA plot for the GI-ME-N models (Supplementary figure 8B) we observe separation between wildtype clones 1+2 and wildtype clones 3+4 especially on PCA1, which explains the largest proportion of the variance. This clearly indicated a batch effect and therefore we compared the GIMEN ATRX aberrant clones with the batch corresponding wild-type clones. To exclude that the difference in ribosome biogenesis gene signatures between the different models was due to the heterogeneity present in wildtype GI-ME-N cells we also conducted the RNA analysis and GSEA for the distinct isogenic GI-ME-N models using all four GI-ME-N wild-type clones. This GSEA also showed ribosome biogenesis among the enriched gene sets (again down in ATRXΔ2-10 and up in ATRXΔ2-13 and knock-out). Nevertheless, the batch effect could have influence on other terms or single genes and therefore we needed to correct for this in all our analyses. Lastly, we now included supplementary figure 15A in main figure 4F.

***Reviewer 3***

Major comments 2. In Figure 3, the authors compare the differential gene expression and gene ontology analysis with ATRX deletion conditions. The authors should do the gene set enrichment analysis/Gene ontology term with WT vs ATRX KO or ATRXΔ2-10 or ATRXΔ2-13 and see whether the ribosome biogenesis pathway shows up. It is unclear from Figure 4B-E why authors have used two different cell lines for GO term comparison as their genetic background is different.

If we understand the reviewer correctly, the reviewer wants us to determine the differentially expressed genes (DEGs) by comparing wildtype versus the distinct ATRX mutant. This is what we already performed as DEGs are determined by comparing WT versus the distinct ATRX mutant. To make this clearer we included a new figure explaining how DEGs are determined in figure 3. Similarly, if we understand the reviewer correctly, the reviewer suggests us to perform gene set enrichment analysis (GSEA) by comparing the WT versus the different mutants, this we already did as GSEA is performed on the stat values (a value that both represents the fold change and significance and is advised for GSEA. These stat values are acquired by performing DEG analysis on wildtype versus mutant). An overview of how we used the DEG lists for GSEA is shown in figure 4A. For the last comment, we added a new sentence explaining why we compared two different cell lines for GO term analysis (page 11) in Figures 4B-E. This we specifically did because of the different genetic background, as we are only interested in DEGs that always change in ATRX aberrant models irrespective of their (epi)genetic background. The changes in expression of those overlapping genes are more likely the direct result of the ATRX aberrations.

***Reviewer 3***

Part of major comment 6. Statistical analyses is missing from almost every Figure.

This was also mentioned by reviewer 1. For the FACS measurements, we performed additional experiments and add the statistical analysis. For figure 5 the p-values and statistics were mentioned in the result section, but we now also added this information to the figure legend.

***Reviewer 3***

Minor comment 1. The immunofluorescence labeling text in the supplementary Figures is not visible. The imaging should be done with confocal microscopy to avoid the background signal and to get a better resolution.

We changed these images and improved the labeling text. Additionally, we show a zoom-in for the merged figures to increase the interpretability. We decided not to perform confocal microscopy as we also performed telomere southern blots which are in agreements with our microscopy pictures.

***Reviewer 3***

Minor comment 2. Please put the appropriate color symbol in supplementary Figure 12A. Currently, the color symbol in the Figure panel does not match the Figure.

We understand that the current labeling of the ATRX status using circles might have led to confusion to the reviewer, therefore we changed the depiction of the ATRX status and also changed the order of the two legends.

## 4. Description of analyses that authors prefer not to carry out

***Reviewer 2***

Minor comment 2. RNA-Seq data for many cancers are now available, and so the authors could perform RNA-Seq analysis across ATRX mutant tumors and correlate with the type of ATRX mutation to see if the dichotomy they observed is present in patient data. This could be done for neuroblastoma and other tumors. The authors state that other tumors do not typically contain multi-exon deletions, but the effect of point mutations on the ATRX protein could similarly be non-uniform.

This is a nice suggestion but is beyond the scope of this study. Our manuscript already contains RNA sequencing data from neuroblastoma tumours (iTHER data), where we find decreased ribosome biogenesis for the two iTHER patients with an exon 2-10 deletion compared to ATRX wild-type neuroblastoma tumors. Nonsense mutations are rare in neuroblastoma, only ~20 patients with such a mutation have been reported in the literature, and our iTHER cohort does not contain any neuroblastoma tumors with *ATRX* nonsense mutations. More extensive analyses across tumor types might be difficult since many RNA-sequencing data sets only contain a few *ATRX* aberrant tumors (and combining distinct data sets is very challenging due to potential batch effects) and for the majority of the rare point mutations (nonsense and missense) and rare deletions no (RNA) sequencing data is available and therefore there will not be enough statistical power.

***Reviewer 3***

Major comments 3. The ribosome biogenesis pathway is up-regulated in the ATRXΔ2-13 model. It is better to test their hypothesis in mice with a xenograft model with WT and ATRXΔ2-13 cell lines in combination with Pol I inhibitor or other well-known drugs which will inhibit the ribosome biogenesis and determine the effects on the growth of the tumor.

This is an interesting suggestion for a follow-up study but would take too much time to perform within the scope of this revision. Additionally, it would be of limited added value to the patients as only two patients with an ATRXΔ2-13 have been reported world-wide. Lastly, the most commonly used RNA polymerase I inhibitor Pidnarulex was recently discovered to inhibit topoisomerase 2B (TOP2B) instead of RNA polymerase 1 (DOI:[10.1038/s41467-021-26640-x](https://www.nature.com/articles/s41467-021-26640-x)).

***Reviewer 3***

Major comments 5. As the ribosome biosynthesis was increased in ATRX KO/ ATRXΔ2-13 compared to ATRXΔ2-10. ATRXΔ2-13 deletion was generated only in GI-ME-N cell line model. To bypass the cell line-specific effect, it is necessary to prepare ATRXΔ2-13 deletion in other cell lines and validate whether the ribosome biogenesis pathway is still induced in another cell line.

Originally, we attempted to also create the ATRXΔ2-13 model in the NB139 cell line (we screened more than 70 clones, which were all wild-type), however generating such large deletion is extremely difficult as the efficiency is very low (several 100 kbs have to be removed). It would take too much time to generate such clones for the revision. Additionally, as mentioned above this ATRX aberration is less relevant to patients as it is very rare. However, the reviewer does have a good point about this limitation and therefore we have included a part in the discussion regarding this limitation (page 18, included in the new paragraph).

***Reviewer 3***

Minor comments 3. The WT GI-ME-N clone should be consistent in all supplementary western blots.

The wild-types samples are only included in the western blots as a positive control and as reference to compare the mutant with. Re-performing all those western blots with the samen WT GI-M-EN would not lead to any changes in the conclusions. Therefore, we think it is not of added value to repeat these western blots.
